# Lipocalin-2 as a prognostic marker in patients with acute exacerbation of idiopathic pulmonary fibrosis

**DOI:** 10.1186/s12931-024-02825-y

**Published:** 2024-05-04

**Authors:** Hiroki Tanahashi, Hiroshi Iwamoto, Kakuhiro Yamaguchi, Shinjiro Sakamoto, Yasushi Horimasu, Takeshi Masuda, Taku Nakashima, Shinichiro Ohshimo, Kazunori Fujitaka, Hironobu Hamada, Noboru Hattori

**Affiliations:** 1 Department of Molecular and Internal Medicine, Graduate School of Biomedical and Health Sciences, Hiroshima University, 1-2-3 Kasumi, Minami-ku, Hiroshima, 734-8551 Japan; 2 Department of Emergency and Critical Care Medicine, Graduate School of Biomedical and Health Sciences, Hiroshima University, Hiroshima, Japan; 3 Department of Physical Analysis and Therapeutic Sciences, Graduate School of Biomedical and Health Sciences, Hiroshima University, Hiroshima, Japan

**Keywords:** Lipocalin-2, Idiopathic pulmonary fibrosis, Oxidative stress, Acute exacerbation

## Abstract

**Background:**

Lipocalin-2 (LCN2) is a secretory glycoprotein upregulated by oxidative stress; moreover, patients with idiopathic pulmonary fibrosis (IPF) have shown increased LCN2 levels in bronchoalveolar lavage fluid (BALF). This study aimed to determine whether circulatory LCN2 could be a systemic biomarker in patients with IPF and to investigate the role of LCN2 in a bleomycin-induced lung injury mouse model.

**Methods:**

We measured serum LCN2 levels in 99 patients with stable IPF, 27 patients with acute exacerbation (AE) of IPF, 51 patients with chronic hypersensitivity pneumonitis, and 67 healthy controls. Further, LCN2 expression in lung tissue was evaluated in a bleomycin-induced lung injury mouse model, and the role of LCN2 was investigated using LCN2-knockout (LCN2 -/-) mice.

**Results:**

Serum levels of LCN2 were significantly higher in patients with AE-IPF than in the other groups. The multivariate Cox proportional hazards model showed that elevated serum LCN2 level was an independent predictor of poor survival in patients with AE-IPF. In the bleomycin-induced lung injury mouse model, a higher dose of bleomycin resulted in higher LCN2 levels and shorter survival. Bleomycin-treated LCN2 -/- mice exhibited increased BALF cell and protein levels as well as hydroxyproline content. Moreover, compared with wild-type mice, LCN2-/- mice showed higher levels of circulatory 8-isoprostane as well as lower Nrf-2, GCLC, and NQO1 expression levels in lung tissue following bleomycin administration.

**Conclusions:**

Our findings demonstrate that serum LCN2 might be a potential prognostic marker of AE-IPF. Moreover, LCN2 expression levels may reflect the severity of lung injury, and LCN2 may be a protective factor against bleomycin-induced acute lung injury and oxidative stress.

**Supplementary Information:**

The online version contains supplementary material available at 10.1186/s12931-024-02825-y.

## Background

Idiopathic pulmonary fibrosis (IPF) is a progressive lung fibrotic disease, and acute exacerbation (AE) of IPF is potentially fatal [1, 2]. There are considerable variations in the clinical course of IPF, with 4.8%–14.2% of patients with IPF developing AE within 1 year [3, 4]. Although the causes of IPF and AE-IPF remain unclear, reactive oxygen species (ROS) have been suggested to be associated with the pathogeneses of IPF and AE-IPF [5–7]. Patients with IPF have increased oxidative products and decreased antioxidants in the lung and circulation [8–10]. Moreover, patients with AE-IPF have higher oxidative stress levels than those with stable IPF [7], and there are significant positive correlations of the AE-IPF incidence with exposure to O_3_, NO_2_, radiation, and particulate matter, which induce oxidative stress [11–13]. However, the regulatory mechanism of oxidative stress in patients with IPF and AE-IPF remains unclear.

Lipocalin-2 (LCN2) is a 25-kDa secreted protein expressed in epithelial cells and immune cells such as neutrophils and macrophages [14]. LCN2 expression is upregulated by exposure to ROS, including O_3_ and H_2_O_2_, and radiation in vitro and in vivo [15, 16]. Additionally, LCN2 has been shown to induce antioxidants in vitro [17]. Elevated LCN2 expression has been observed in patients with chronic obstructive pulmonary disease and acute respiratory distress syndrome, which have been associated with increased oxidative stress levels [18–20]. Moreover, Ikezoe et al. reported that patients with IPF had higher LCN2 levels in bronchoalveolar lavage fluid (BALF) than those with other interstitial pneumonia [21]. However, it remains unclear whether LCN2 could be a systemic biomarker in patients with IPF; further, the association of LCN2 with the pathogenesis of IPF and AE-IPF remains to be elucidated.

This study aimed to investigate whether circulatory LCN2 could be a biomarker of IPF. Further, we aimed to examine lung and circulatory LCN2 levels in a bleomycin (BLM)-induced lung injury mouse model. Finally, we aimed to investigate the role of LCN2 in BLM-induced lung injury using LCN2-knockout (LCN2 -/-) mice.

## Materials and methods

### Study population and design

This study included 99 patients with stable IPF who were followed up for >6 months, 27 patients with AE-IPF, 51 patients with chronic hypersensitivity pneumonitis (CHP), and 67 healthy controls. The patients with IPF and CHP were diagnosed at Hiroshima University Hospital between June 2002 and June 2020. IPF was diagnosed following the American Thoracic Society (ATS)/European Respiratory Society (ERS) criteria [1]. Patients with stable IPF were defined as patients with IPF who are stable for 2 months before the examination [22]. The patients with AE-IPF were diagnosed using the International Working Group Report diagnostic criteria for AE-IP [3]. CHP was diagnosed according to criteria for CHP proposed by Yoshizawa et al [23]. This study was approved by the Ethics Committee of Hiroshima University Hospital (E2004-0326 and E2023-0185); further, all participants provided written informed consent.

### Animals

LCN2-/- mice (backcrossed for at least 10 generations to C57BL/6J mice) were purchased from The Jackson Laboratory (Bar Harbor, ME, USA) and bred. The lack of LCN2 was confirmed through polymerase chain reaction (PCR) analysis (Figure S1). Wild-type (WT) female C57BL/6 mice (Charles River Japan Inc., Yokohama, Japan) and LCN-/- mice were housed in the pathogen-free environment at an optimal temperature with a 12-h light/dark cycle; moreover, they were used for experiments at the age of 9–10 weeks. All protocols were approved by the Committee on Animal Research at Hiroshima University (Approval No. A21-121, 19-123) and were conducted under the Guide for the Care and Use of Laboratory Animals, 8th ed, 2010 (National Institutes of Health, Bethesda, MD, USA).

### Establishment of the BLM-induced lung injury mouse model

On day 0, the mice were first anesthetized with mixed anesthetic agents, including medetomidine (Kyoritsu Seiyaku, Tokyo, Japan), midazolam (Sandoz K.K., Tokyo, Japan), and butorphanol (Meiji Seika Pharma, Tokyo, Japan). Subsequently, the mice were intraorally administered with BLM (Nippon Kayaku, Tokyo, Japan) at 2.0 mg/kg and 10.0 mg/kg body weight in phosphate-buffered saline (PBS) via oropharyngeal aspiration.

### Mouse bronchoalveolar lavage fluid

The trachea was exposed and cannulated with an 18-gauge cannula, and the lungs were lavaged three times using 0.5 ml of PBS. The lavage fluids were pooled and centrifuged at 1500 rpm for 5 min at 4°C. The supernatants were stored at -80°C prior to measurement of protein and LCN2 levels. The cell pellets were resuspended in 1 ml of Dulbecco's Modified Eagle Medium, and the total cell numbers were counted using an automated cell counter. Differential cell counts were determined by counting at least 300 cells on a smear prepared using a cytospin (Thermo Fisher Scientific, USA) and stained with the Diff-Quik stain (Kokusai Shiyaku, Kobe, Japan).

### Measurement of LCN2, 8-isoprostane, and protein concentrations

Human blood samples were collected and stored at -80°C until subsequent analysis. Serum LCN2 levels and mice BALF were measured using commercially available enzyme-linked immunosorbent assay kits following the manufacturer’s instructions [human and mouse LCN2 Quantikine ELISA kits (R&D Systems, Minneapolis, MN)]. The mice BALF protein levels were determined using a BCA protein assay kit (Pierce, Rockford, IL, USA) following the manufacturer's instructions. Mice serum was analyzed using the 8-Isoprostane ELISA Kit (Cayman Chemical, Inc.) according to the manufacturer's protocol.

### Hydroxyproline assay

Mice lungs were evaluated for hydroxyproline content as previously described [24]. Briefly, the sample was homogenized in 1 mL of PBS and hydrolyzed using 1 mL of HCl. The sample was centrifuged, and the supernatant was used for hydroxyproline assay. Citrate/acetate buffer (deionized distilled water supplemented with 238 mM Citric acid, Sigma-Aldrich; 1.2% glacial acetic acid, Sigma-Aldrich; 532 mM sodium acetate, Sigma-Aldrich; and 850 mM sodium hydroxide, Nacalai Tesque) and chloramine T solution (1.0 mL deionized distilled water supplemented with 0.141 g chloramine T, Sigma-Aldrich; 1.0 mL 1-propanol, Sigma-Aldrich; and 8.0 mL citrate/acetate buffer) were added. After incubation, Ehrlich’s reagent (2.5 g 4-dimethylaminobenzaldehyde, Sigma-Aldrich; 9.3 mL 1-propanol and 3.9 mL 70% perchloric acid; Sigma-Aldrich) was added. After incubation at 65°C for 30 minutes, the absorbance was measured using a plate reader (iMARK, Bio-Rad, Hercules, CA, USA).

### PCR analysis

Total RNA was extracted from mice lungs using the RNeasy Mini Kit (QIAGEN, Venlo, Netherlands). The RNA was reverse transcribed into cDNA using the High-Capacity RNA-to-cDNA Kit (Applied Biosystems, Foster City, CA, USA). Real-time quantitative PCR was performed using the Applied Biosystems 7500 Fast Real-Time PCR System (Applied Biosystems) and the TaqMan Gene Expression Assays (Applied Biosystems) as previously described [25]. The expression of Actb (β-actin, Mm02619580_g1; Applied Biosystems) was used as an internal control. The TaqMan Gene Expression Assays were used as LCN2 (Lcn2, Mm01324470_m1), GCLC (Gclc, Mm00802658_m1), Nrf2 (Nfe2l2, Mm00477784_m1), and NQO1(Nqo1, Mm01253561_m1; Applied Biosystems).

### Lung histology and immunohistochemical staining

Mouse lungs were fixed in 4% formalin and embedded in paraffin. The lung sections were stained with hematoxylin and eosin. Immunohistochemical staining for LCN2 was performed using Dako EnVison+ System-HRP (DAB) (Dako, Tokyo, Japan) following the manufacturer’s instructions. Anti-LCN2 antibody (ab216462, Abcam, Cambridge, UK) was used as primary antibody.

### Statistical analysis

Values are expressed as medians (interquartile ranges [IQRs]), unless stated otherwise. Among-group comparisons were performed using Kruskal-Wallis tests, followed by Mann-Whitney *U* tests and Pearson’s chi-squared tests. Age-adjusted multivariate linear regression analyses, followed by Holm’s correction, were used to examine differences in serum LCN2 levels between healthy controls and patients with CHP, stable IPF, or AE-IPF. Continuous data at two time points in the same patient were compared using the Wilcoxon signed-rank test. Receiver operating characteristic (ROC) curve analysis was performed to determine the optimal serum LCN2 cut-off levels. Survival and AE development was analyzed using Kaplan-Meier analysis and log-rank test. Cox proportional hazards analysis was used to examine the prognosis of patients with AE-IPF. All *P*-values < 0.05 were considered statistically significant. All statistical analyses were performed using JMP version 16.0.0 (SAS Institute Inc., Cary, NC, USA).

## Results

### Patients characteristics

The demographic characteristics of the healthy controls as well as patients with stable IPF, AE-IPF, and CHP are shown in Table 1. Patients with AE-IPF had significantly higher white blood cells, neutrophil percentage, and lactate dehydrogenase levels than patients with stable IPF. There were significant among-group differences in age and lung function. The median follow-up durations of the patients with stable IPF and AE-IPF were 42.5 (27.3–62.7) months and 4.21 (0.7-18.0) months, respectively. During the observation period, 35 (35.4%) patients with stable IPF developed AE-IPF.

**Table 1:** Baseline characteristics

	Controls (*n* = 67)	CHP (*n* = 51)	Stable IPF (*n* = 99)	AE-IPF (*n* = 27)	*P*-value
Age, years	57 (56–60)	69 (63–74)	68 (61–74)	69 (62–76)	<0.001 *
Sex					0.005 *
male, n (%)	56 (83.6)	32 (62.7)	86 (86.9)	21 (77.8)	
female, n (%)	11 (16.4)	19 (37.3)	13 (13.1)	6 (22.2)	
Smoking history, pack-years^a^	21.3 (0–42.8)	7 (0–31)	33 (15–48)	35 (12.2–45.8)	<0.001*
WBC (/μL)	5300 (4500–7090)	6740 (5700–8400)	6880 (5740–8050)	11250 (8380–14400)	<0.001*
Neutrophil (%)^b^		60.3 (53.7–65.3)	60.1 (55.3–66.8)	80.6 (69.0–86.0)	<0.001*
LDH (U/L)		222 (201–255)	216 (192–236)	356 (269–479)	<0.001*
CCr (mL/min)^c^	87.2 (75.4–98.2)	74.6 (61.1–92.1)	76.5 (61.9–93.3)	58.0 (50.2–86.2)	<0.001*
CRP (mg/dL)^d^	0.07 (0.03–0.19)	0.21 (0.1–0.48)	0.13 (0.07–0.33)	7.4 (2.0–10.2)	<0.001*
FVC (%predicted)^e^	101.3 (91.9–110.9)	76.3 (60.6–92.2)	70.5 (60.9–86.1)		<0.001*
DLCO (%predicted)^f^		44.1 (37.0–59.1)	47.1 (36.6–59.3)		
GAP staging system, I / II / III /unknown		21/ 16/ 5/ 9	37/ 40/ 12/ 10		
LCN2 (ng/mL)	42.9 (27.6–67.7)	68.1 (53.5–88.3)	73.5 (56.1–102.3)	118.4 (86.1–181.9)	<0.001*

Data are presented as median and interquartile range (IQR) unless stated otherwise

*CHP* Chronic hypersensitivity pneumonitis, *IPF* Idiopathic pulmonary fibrosis, *AE* Acute exacerbation, *IQR* Interquartile range, *WBC* White blood cell, *LDH* Lactate dehydrogenase, *CCr* Creatinine clearance, *CRP* C-reactive protein, *FVC* Forced vital capacity, *DLCO* Diffusion lung capacity for carbon monoxide, *GAP* Gender, age, and physiology

^a^Smoking history was missing in one patient with AE-IPF and three healthy controls

^b^Neutrophil was available for 50 patients with CHP

^c^CCr was available for 25 patients with AE-IPF

^d^CRP was available for 66 healthy controls

^e^FVC values were available for 97 and 50 patients with stable IPF and CHP, respectively

^f^DLCO values were available for 89 and 42 patients with stable IPF and CHP, respectively

^*^P < 0.05, comparison among three or four groups via Kruskal-Wallis test or Pearson's chi-square test unless otherwise stated

### Elevated serum levels of LCN2 in patients with AE-IPF

**Fig. 1:**
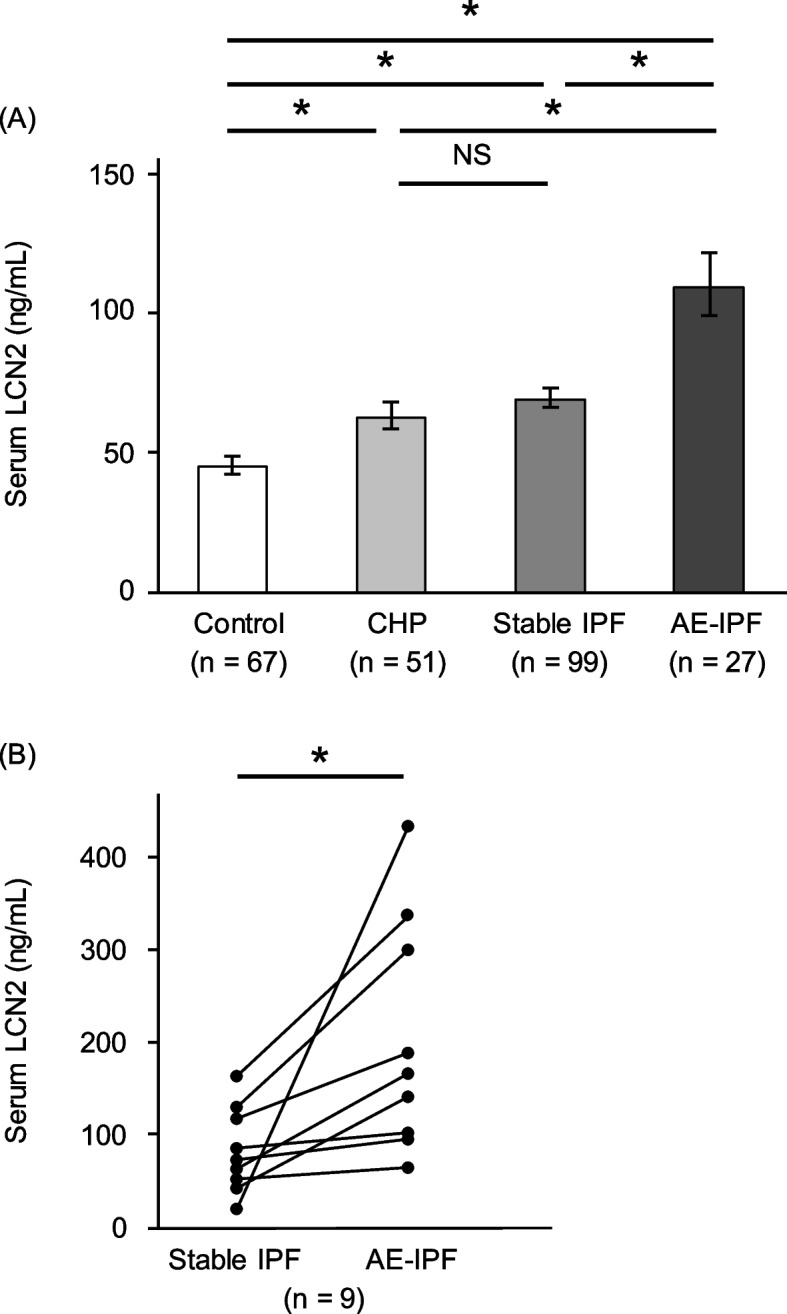
Comparison of serum LCN2 levels. Comparison of serum LCN2 levels among healthy controls and patients with CHP, stable IPF, and AE-IPF was examined by multivariate linear regression analyses, followed by Holm’s correction after adjusting for age. Data are presented as backtransformed least squares mean ± standard errors (**A**). Serum LCN2 levels were significantly increased during the AE phase than in the stable phase in longitudinal analysis of nine patients with IPF (73.5 ng/mL [46.8–123.8] vs 167.1 ng/mL [99.1–318.1], *P* = 0.004) (**B**). *P < 0.05 using multivariate linear regression analyses, followed by Holm’s correction or the Wilcoxon signed-rank test. LCN2, lipocalin-2; CHP, chronic hypersensitivity pneumonitis; IPF, idiopathic pulmonary fibrosis; AE, acute exacerbation; NS, not significant

We compared serum LCN2 levels between healthy controls and patients with CHP, stable IPF, and AE-IPF by age-adjusted multivariate linear regression analyses, followed by Holm’s correction (Fig. 1A). Serum LCN2 levels in patients with stable IPF or CHP were significantly higher than those in controls (*P* < 0.001, *P* = 0.001). There was no significant difference in serum LCN2 levels between patients with stable IPF and those with CHP. Serum LCN2 levels in patients with AE-IPF were significantly higher than those in the other groups (*P* < 0.001). Additionally, longitudinal analysis showed that serum LCN2 levels were significantly increased from stable phase to AE phase in nine patients (73.5 ng/mL [46.8–123.8] vs. 167.1 ng/mL [99.1–318.1], *P* = 0.004; Fig. 1B).

Serum LCN2 levels were positively correlated with baseline age, white blood cells, neutrophil percentage, and C-reactive protein levels (Table S1). Contrastingly, serum LCN2 levels were negatively correlated with creatinine clearance. Lung function was not associated with serum LCN2 levels in patients with stable IPF and CHP.

### Association of serum LCN2 levels with survival in AE-IPF

**Fig. 2:**
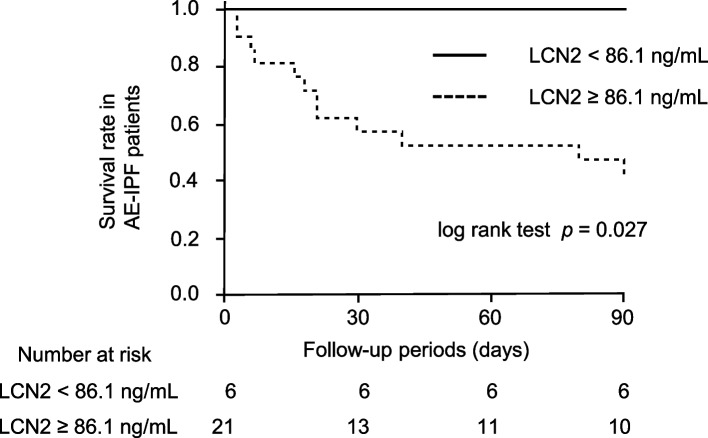
Kaplan-Meier analysis for 3-month survival in AE-IPF. The 3-month survival rate was significantly lower in patients with AE-IPF with higher serum LCN2 levels (≥86.1 ng/mL) than in patients with lower LCN2 (<86.1 ng/mL) (median survival time, higher group; 80 days vs. lower group; not reached, log-rank test; *P* = 0.027). AE, acute exacerbation; IPF, idiopathic pulmonary fibrosis; LCN2, lipocalin-2

ROC analysis revealed that serum LCN2 levels had an area under the curve of 0.628 for predicting 3-month survival in patients with AE-IPF (Figure S2). A cut-off value of 86.1 ng/mL provided 100.0% sensitivity and 40.0% specificity. When patients were divided into two groups based on this cut-off level, the 3-month survival rate was significantly lower in patients with high LCN2 levels than in patients with low levels (Fig. 2; log-rank test, *P* = 0.027). A multivariate Cox proportional hazards model revealed that higher levels of serum LCN2 were significantly associated with poor survival after adjusting for background factors, including age, sex, smoking history, inflammatory markers, and creatinine clearance (Table 2).

**Table 2:** Cox proportional hazards model for 3-month survival in patients with AE-IPF

Variables	HR	95%CI	*P*-value
Univariate analysis			
Age, years	1.007	0.948–1.070	0.810
Sex, male	0.863	0.257–3.894	0.830
Smoking history, pack-years	1.010	0.982–1.040	0.471
WBC (/μL)	1.000	0.999–1.000	0.051
Neutrophil (%)	1.066	1.007–1.142	0.025
LDH (U/L)	1.003	0.999–1.007	0.080
CCr (mL/min)	0.972	0.934–1.001	0.062
CRP (mg/dL)	1.050	0.978–1.119	0.169
LCN2 ≥ 86.1 ng/mL	not available^a^	not available^a^	0.004
Multivariate analysis			
LCN2 ≥ 86.1 ng/mL	not available^a^	not available^a^	0.019
Neutrophil (%)	1.053	0.987–1.131	0.121

*AE* Acute exacerbation, *IPF* Idiopathic pulmonary fibrosis, *HR* Hazards ratio, *CI* Confidence interval, *WBC* White blood cell, *LDH* Lactate dehydrogenase, *CCr* Creatinine clearance, *CRP* C-reactive protein, *LCN2* Lipocalin-2

^a^HR and 95%CI could not be calculated because there was no mortality in the low LCN2 (< 86.1 ng/mL) group

There was no significant association between the optimal cut-off level of serum LCN2 and survival (data not shown) in patients with stable IPF. A trend of earlier AE onset was found in patients with higher LCN2 levels (≥ 106.3 ng/mL) than in those with lower LCN2 levels (*P* = 0.096; Figure S3).

### Expression and localization of LCN2 in BLM-induced lung injury mice

**Fig. 3:**
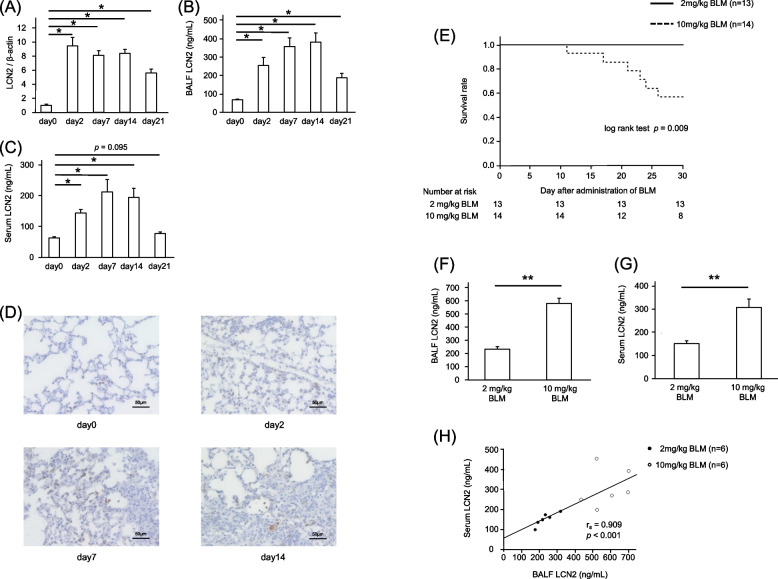
LCN2 expression in BLM-induced lung injury model**.** Expression of LCN2 mRNA was measured in mice lungs on day 0 (before BLM administration) and on days 2, 7, 14, and 21 after BLM administration (BLM 2mg/kg, *n* = 5–6 per group) (**A**). BALF and blood concentrations of LCN2 at day 0 (before BLM administration) and at days 2, 7, 14, and 21 after BLM administration (*n* = 5–6 per group) (**B, C**). Representative images of immunohistochemical staining of LCN2 in the lung were obtained on day 0 (before BLM administration) and days 2, 7, and 14 after BLM administration (**D**). Kaplan-Meier analysis for 1-month survival in BLM-induced lung injury model mice with a BLM dose of 2 mg/kg or 10 mg/kg (**E**). BALF and blood concentrations of LCN2 at day 2 in mice with a BLM dose of 2 mg/kg and 10mg/kg (*n* = 6 per group) (**F, G**). The correlation between BALF and circulatory LCN2 levels at day 2 in BLM-induced lung injury mice (**H**). Data are presented as mean ± SEM. *P < 0.05, ** *P* < 0.01 using the Mann-Whitney U test. Scale bar = 50 μm. LCN2, lipocalin-2; BLM, bleomycin; BALF, bronchoalveolar lavage fluid; SEM, standard error of the mean

In the BLM-induced lung injury model, mRNA levels of LCN2 in the lung increased after BLM from day 2 to day 21 (Fig. 3A). Additionally, BALF and serum LCN2 levels were increased from day 2 to day 21 (Fig. 3B, C). Immunohistochemical staining showed positive LCN2 expression in inflammatory cells in both the central and subpleural lung regions, as well as in the fibrotic interstitium in BLM-induced lung injury mice (Fig. 3D).

A higher BLM dose (10 mg/kg) resulted in higher mortality as well as higher BALF and circulatory LCN2 levels at day 2 (Fig. 3E–G). There was a significant positive correlation between BALF and circulatory LCN2 levels at day 2 in BLM-induced lung injury mice (Fig. 3H).

### BLM administration increased the severity of lung fibrosis as well as BALF cells and proteins in LCN2-/- mice

**Fig. 4:**
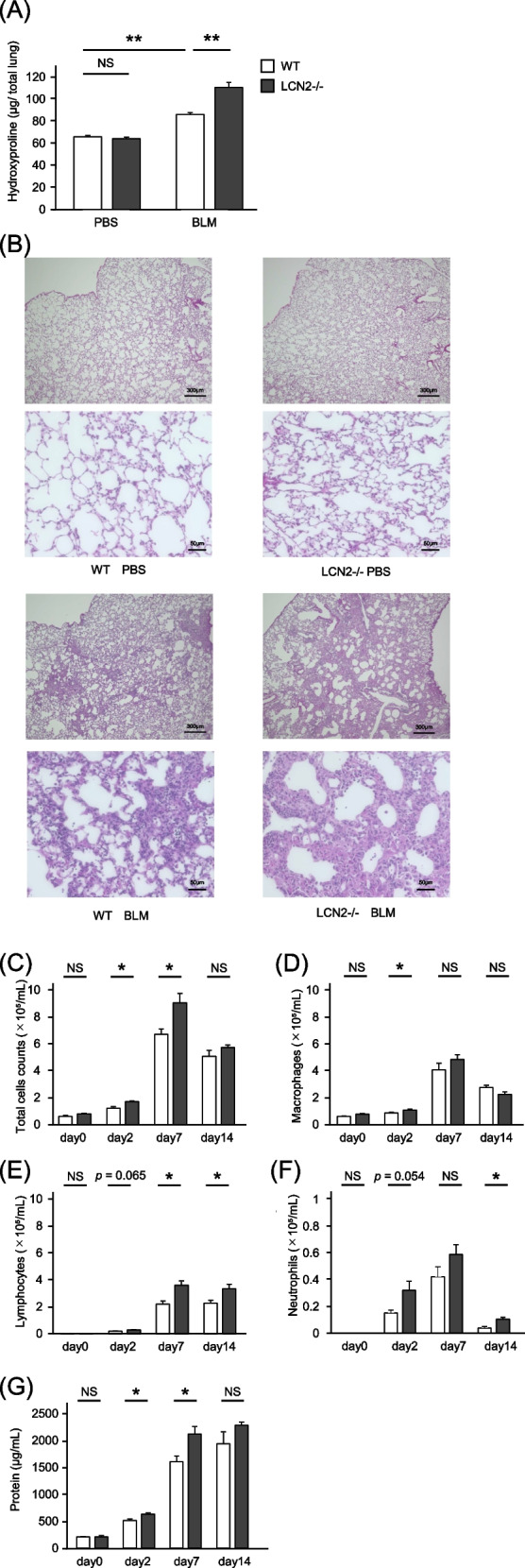
Enhanced lung injury in LCN2 knockout mice by BLM administration. Hydroxyproline concentration was measured on day 14 after administration of 2 mg/kg BLM or PBS (*n* = 6–8 per group) (**A**). Representative images of hematoxylin and eosin staining in the lung section on day 14 after BLM or PBS administration (**B**). BALF analysis on day 0 (before BLM administration) and days 2, 7, 14, and 21 (*n* = 5–7 per group) (**C**–**G**). Data are shown as mean ± SEM. WT mice (open bars) or LCN2-/- mice (solid bars). *P < 0.05, ** *P* < 0.01 using the Mann-Whitney U test. Scale bar = 300 or 50 μm. LCN2, lipocalin-2; BLM, bleomycin; PBS, phosphate-buffered saline; BALF, bronchoalveolar lavage fluid; NS, not significant; WT, wild type; SEM, standard error of the mean

BLM-treated LCN2-/- mice showed significantly higher hydroxyproline content than BLM-treated WT mice (Fig. 4A); however, the hydroxyproline content did not significantly differ between WT and LCN2 -/- mice before BLM administration. Hematoxylin and eosin staining of lung tissue showed greater alveolar wall thickening and extracellular matrix deposition in BLM-treated LCN2-/- mice than in BLM-treated WT mice (Fig. 4B). Moreover, the total cell counts and protein levels in the inflammatory phase of BALF were higher in LCN2-/- mice than in WT mice on days 2 and 7 after BLM administration (Fig. 4C–G).

### Reduced antioxidative pathway in LCN2 -/- mice by BLM administration

**Fig. 5:**
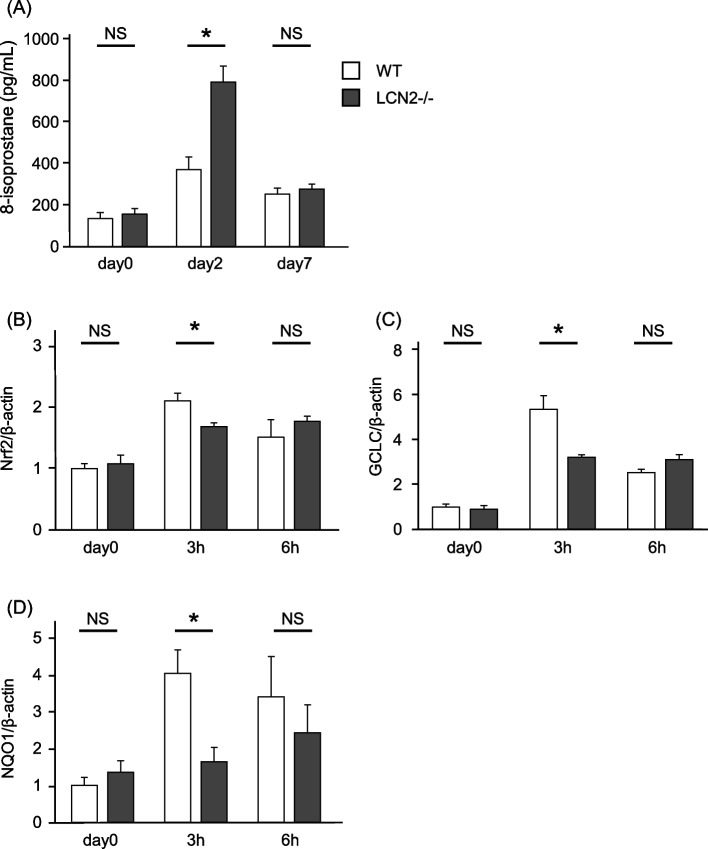
Reduced antioxidative response in LCN2 knockout mice after BLM administration. The serum 8-isoprostane was measured at day 0 (before 2 mg/kg BLM administration) and days 2 and 7 (n = 5–6 per group) (**A**). The expression of Nrf2, GCLC, and NQO1 mRNAs in the lungs of WT mice or LCN2-/- mice at baseline and 3 and 6 hours after BLM administration (**B, C**, and **D**). β-actin was used as an endogenous control (*n* = 5–6 per group). WT mice (open bars) or LCN2-/- mice (solid bars). Data are shown as mean ± SEM. *P < 0.05 using the Mann-Whitney U test. LCN2, lipocalin-2; BLM, bleomycin; WT, wild type; NS, not significant; Nrf2, NF-E2-related factor 2; GCLC, glutamate-cysteine ligase catalytic; NQO1, quinone 1; SEM, standard error of the mean

We assessed the degree of oxidative stress and antioxidants in WT and LCN2-/- mice to investigate the pathophysiological role of LCN2 in BLM-induced lung injury. The serum levels of 8-isoprostane were elevated in BLM-treated LCN2-/- mice than in BLM-treated WT mice after 2 days (Fig. 5A). Next, we examined the mRNA expression of Nrf2, a transcription factor for antioxidant stress response, and its downstream mRNAs, GCLC, and NQO1 in the mouse lungs. The expression levels of these antioxidants in the lungs were significantly lower in LCN2-/- mice than in WT mice at 3 hours after BLM administration (Fig. 5B–D). Before BLM administration, there was no significant difference in circulatory 8-isoprostane and lung expression levels of Nrf2, GCLC, and NQO1 between WT and LCN2-/- mice.

## Discussion

This study demonstrated that serum LCN2 levels were elevated in patients with AE-IPF, which was associated with poor survival. LCN2 was upregulated in BLM-induced lung injury mice; further, the BLM dose was positively and negatively correlated with LCN2 expression and survival, respectively. Notably, BLM-treated LCN2 -/- mice showed enhanced lung inflammation and fibrosis compared with BLM-treated WT mice. Moreover, LCN2 -/- mice showed higher levels of circulatory 8-isoprostane and lower expression of Nrf-2, GCLC, and NQO1 levels than WT mice. Our results demonstrate that serum levels of LCN2 could be a prognostic biomarker of AE-IPF. Additionally, the results of the BLM-induced mouse model indicate that higher LCN2 expression might be an indicator of more severe lung injury, with LCN2 potentially providing protection against lung injury and oxidative stress.

This study demonstrated that elevated levels of serum LCN2 are independently associated with poor survival in patients with AE-IPF after adjustment for background factors. Moreover, BLM-induced lung injury mice showed upregulated LCN2 levels in lung tissue and circulation, with a higher BLM dose resulting in higher LCN2 expression and shorter survival. Previous studies have shown that increased ROS is associated with the pathogenesis underlying AE-IPF and BLM-induced lung injury in mouse models [7, 11, 12, 26]. Moreover, increased oxidative stress induced by radiation or ozone exposure has been shown to upregulate LCN2 expression in lung epithelial cells in vitro and in vivo [15, 16, 27]. Roudkenar et al. demonstrated that H_2_O_2_ upregulates LCN2 expression in vitro, which is reversed by the addition of antioxidants [15]. These findings indicate that LCN2 is a potential biomarker for detecting oxidative stress. Moreover, elevated LCN2 levels were found to be associated with poor survival in several diseases, including cancer and stroke [28, 29]. Taken together, higher LCN2 expression may reflect more severe lung injury; further, serum LCN2 is a potential prognostic biomarker in patients with AE-IPF.

In our study, BLM-treated LCN2 -/- mice exhibited increased BALF inflammatory cells and circulatory 8-isoprostane levels compared with BLM-treated WT mice. Moreover, Nrf2 and its downstream molecules, GCLC and NQO1, were upregulated in BLM-treated WT mice. There was no difference in lung fibrosis, BALF inflammatory cells, and oxidative stress/antioxidant-related molecules between LCN2-/- and WT mice before BLM administration. These results indicate that BLM administration upregulated LCN2, which exerts protective effects against oxidative stress, lung inflammation, and fibrosis in the BLM-induced lung injury mouse model. In the model of LPS-induced sepsis, LCN2-/- mice showed higher oxidative products in the liver compared with WT mice [30]. Additionally, LCN2 knockdown in A549 cells was shown to enhance H_2_O_2_ toxicity [31]. Bahmani et al. found that LCN2 expression in CHO and HEK293T cells induces antioxidant expression in vitro [17]. The mechanism underlying the protective effects of LCN2 against oxidative stress remain unclear; however, our findings suggest that LCN2 may induce the Nrf2 antioxidant pathway against BLM-induced lung injury in mice. Previous studies have demonstrated the protective effects of recombinant LCN2 against ischemia-reperfusion kidney injury in vivo and LPS-induced cellular injury in vitro [32, 33]. Since our findings suggest a protective effect of LCN2, further studies are warranted to investigate whether LCN2 can be used as a therapeutic molecule for treating AE-IPF.

This study has limitations. First, this was a small-scale retrospective study; therefore, further large-scale prospective studies are warranted. Second, the function of LCN2 was examined using knockout mice with BLM-induced lung injury models that did not fully represent patients with IPF and AE-IPF. In addition, it is unclear whether LCN2 provides a concentration-dependent antioxidant stress response.

## Conclusion

This study showed that elevated levels of serum LCN2 are an independent risk factor for poor survival in patients with AE-IPF. In BLM-induced lung injury mice, a higher dose of BLM resulted in higher LCN2 expression and shorter survival; further, LCN2 knockout resulted in more severe lung injury, fibrosis, and reduced Nrf2 and its downstream molecules. These results indicated that serum LCN2 might be a potential prognostic marker of AE-IPF. Additionally, higher LCN2 expression levels may reflect the severity of lung injury, and LCN2 may be protective against BLM-induced acute lung injury and oxidative stress.

### Supplementary Information

**Supplementary Material 1.**

## Data Availability

Data are available to interested researchers upon reasonable request to the corresponding author based on ethical approval.

## Abbreviations

IPF: Idiopathic pulmonary fibrosis
AE: Acute exacerbation
ROS: Reactive oxygen species
BALF: Bronchoalveolar lavage fluid
LCN2: Lipocalin-2
CHP: Chronic hypersensitivity pneumonitis
WT: Wild type
BLM: Bleomycin
PCR: Polymerase chain reaction
PBS: Phosphate-buffered saline
IQR: Interquartile range
ROC: Receiver operating characteristic
