# Supplementary material for: Lipocalin-2 as a prognostic marker in patients with acute exacerbation of idiopathic pulmonary fibrosis
Source: Respir Res. 2024 May 4;25:195. doi: 10.1186/s12931-024-02825-y (PMC11070072; doi:10.1186/s12931-024-02825-y)
Supplement: Supplementary file 1 — Supplementary Material 1. [file 12931_2024_2825_MOESM1_ESM.docx]

**Additional file**

**Supplemental Figure S1.** The expression of LCN2 mRNA using PCR analysis


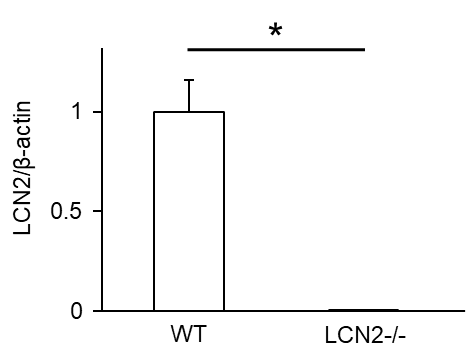


The expression of LCN2 mRNA in the lungs of WT mice or LCN2-/- mice. β-actin was used as an endogenous control (n = 5 per group).

Data are shown as mean ± SEM. * P < 0.05 using the Mann-Whitney U test.

LCN2, lipocalin-2; PCR, polymerase chain reaction; WT, wild type; SEM, standard error of the mean.

**Supplemental Figure S2.** ROC curve analysis of 3-month survival in AE-IPF


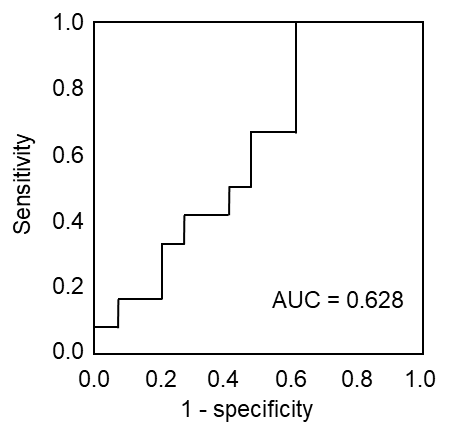


ROC analysis revealed that serum LCN2 levels yielded an area under the curve of 0.628 for predicting 3-month survival. The optimal 86.1 ng/mL cut-off level provided 100.0% sensitivity and 40.0% specificity.

ROC, receiver operating characteristic; AE, acute exacerbation; IPF, idiopathic pulmonary fibrosis; LCN2, Lipocalin-2.

**Supplemental Figure S3.** Serum Lipocalin-2 (LCN2) levels and Kaplan-Meier analysis for the onset of acute exacerbation in stable IPF

**
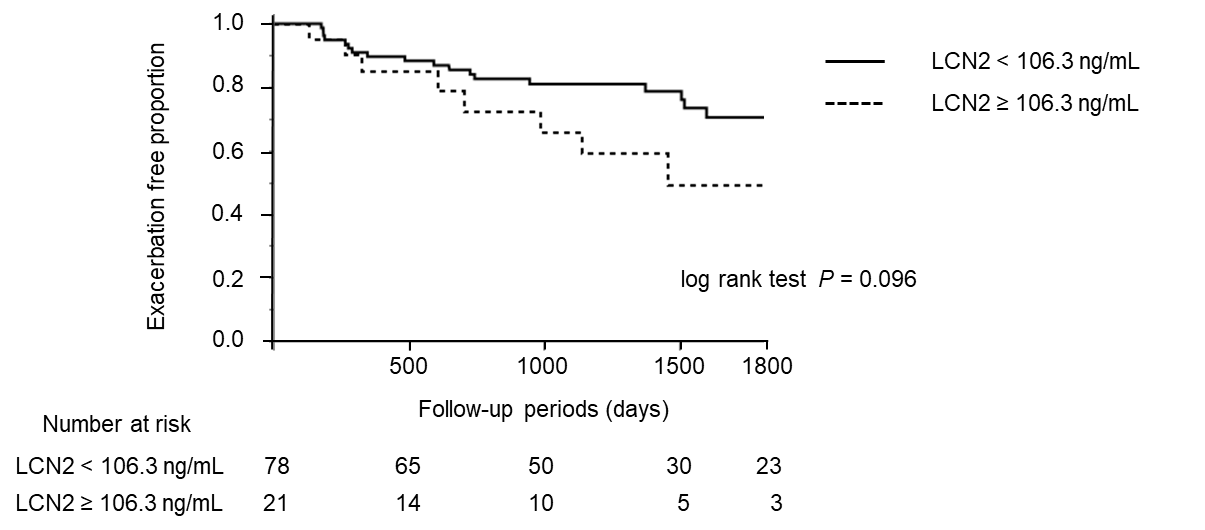
**

Patients with LCN2 level ≥ 106.3 ng/mL had a trend of earlier development of AE compared to those of lower but not significant (log-rank test, P = 0.096).

LCN2, Lipocalin-2; IPF, idiopathic pulmonary fibrosis; AE, acute exacerbation.

**Supplemental Table S1.** Association of Lipocalin-2 levels with patients’ characteristics

|  | Controls (n=67) | | CHP (n=51) | | Stable IPF (n=99) | | AE-IPF (n=27) | |
| --- | --- | --- | --- | --- | --- | --- | --- | --- |
|  | ρ | P-value* | ρ | P-value* | ρ | P-value* | ρ | P-value* |
| Age, years | 0.133 | 0.284 | 0.442 | 0.001 | 0.540 | <0.001 | 0.339 | 0.084 |
| Smoking history,  pack-years | -0.063 | 0.621 | -0.082 | 0.569 | 0.064 | 0.527 | -0.039 | 0.852 |
| WBC, /μL | 0.308 | 0.011 | 0.322 | 0.021 | 0.299 | 0.003 | 0.438 | 0.022 |
| Neutrophil, % |  |  | 0.339 | 0.016 | 0.381 | <0.001 | 0.410 | 0.034 |
| LDH, U/L |  |  | 0.117 | 0.413 | 0.129 | 0.205 | 0.127 | 0.529 |
| CCr, mL/min | -0.094 | 0.451 | -0.271 | 0.055 | -0.273 | 0.006 | -0.303 | 0.141 |
| CRP, mg/mL | 0.279 | 0.023 | 0.249 | 0.078 | 0.308 | 0.002 | 0.376 | 0.053 |
| FVC, %predicted | 0.191 | 0.122 | -0.124 | 0.391 | 0.012 | 0.907 |  |  |
| DLCO, %predicted |  |  | -0.296 | 0.057 | -0.090 | 0.403 |  |  |

* Spearman's rank correlation coefficient

CHP, chronic hypersensitivity pneumonitis; IPF, idiopathic pulmonary fibrosis; AE, acute exacerbation; WBC, white blood cell; LDH, Lactate dehydrogenase; CCr, Creatinine clearance; CRP, C-reactive protein; FVC, forced vital capacity; DLCO, diffusion lung capacity for carbon monoxide.
